# Comparative study of leaf nutrient reabsorption by two different ecotypes of wild soybean under low-nitrogen stress

**DOI:** 10.7717/peerj.15486

**Published:** 2023-06-27

**Authors:** Yuan Liu, Shujuan Gao, Yunan Hu, Tao Zhang, Jixun Guo, Lianxuan Shi, Mingxia Li

**Affiliations:** 1 Northeast Normal University, Changchun, China; 2 ChiFeng University, ChiFeng, China; 3 ChangChun Normal University, Changchun, China

**Keywords:** Wild soybean, Low nitrogen, Metabolomics, Physiological, Nutrient reabsorption

## Abstract

Wild soybean (*Glycine soja*), the ancestor of cultivated soybean, has evolved into many ecotypes with different adaptations to adversity under the action of divergent evolution. Barren-tolerant wild soybean has developed adaptation to most nutrient-stress environments, especially with respect to low nitrogen (LN) conditions. This study describes the differences in physiological and metabolomic changes between common wild soybean (GS1) and barren-tolerant wild soybean(GS2) under LN stress. Compared with plants grown under the unstressed control (CK) conditions, the young leaves of barren-tolerant wild soybean under LN conditions maintained relatively stable chlorophyll, concentration and rates of photosynthesis and transpiration, as well as increased carotenoid content, whereas the net photosynthetic rate (*P*_N_) of GS1 decreased significantly 0.64-fold (*p* < 0.05) in the young leaves of GS1. The ratio of internal to atmospheric CO_2_ concentrations increased significantly 0.07-fold (*p* < 0.05), 0.09-fold (*p* < 0.05) in the young leaves of GS1 and GS2, respectively, and increased significantly 0.05-fold (*p* < 0.05) and 0.07-fold (*p* < 0.05) in the old leaves of GS1 and GS2, respectively, relative to the CK. The concentration of chlorophylls a and *b* decreased significantly 0.45-fold (*p* < 0.05), 0.13-fold (*p* > 0.05) in the young leaves of GS1 and GS2, respectively, and decreased significantly 0.74-fold (*p* < 0.01) and 0.60-fold (*p* < 0.01) in the old leaves of GS1 and GS2, respectively. Under LN stress, nitrate concentration in the young leaves of GS1 and GS2 decreased significantly 0.69- and 0.50-fold (*p* < 0.01), respectively, relative to CK, and decreased significantly 2.10-fold and 1.77-fold (*p* < 0.01) in the old leaves of GS1 and GS2, respectively. Barren-tolerant wild soybean increased the concentration of beneficial ion pairs. Under LN stress, Zn^2+^ significantly increased by 1.06- and 1.35-fold (*p* < 0.01) in the young and old leaves of GS2 (*p* < 0.01), but there was no significant change in GS1. The metabolism of amino acids and organic acids was high in GS2 young and old leaves, and the metabolites related to the TCA cycle were significantly increased. The 4-aminobutyric acid (GABA) concertation decreased significantly 0.70-fold (*p* < 0.05) in the young leaves of GS1 but increased 0.21-fold (*p* < 0.05) significantly in GS2. The relative concentration of proline increased significantly 1.21-fold (*p* < 0.01) and 2.85-fold (*p* < 0.01) in the young and old leaves of GS2. Under LN stress, GS2 could maintain photosynthesis rate and enhance the reabsorption of nitrate and magnesium in young leaves, compared to GS1. More importantly, GS2 exhibited increased amino acid and TCA cycle metabolism in young and old leaves. Adequate reabsorption of mineral and organic nutrients is an important strategy for barren-tolerant wild soybeans to survive under LN stress. Our research provides a new perspective on the exploitation and utilization of wild soybean resources.

## Introduction

The identification and evaluation of germplasm resources is a basic and vital challenge for industrial crop improvement (Abdurakhmonov & Abdusattor, 2008). Wild soybean (*Glycine soja*) is considered the progenitor of cultivated soybean (*Glycine max*) and has been challenged by the natural environment for thousands of years to withstand various biotic and abiotic stresses (Zhang et al., 2017). Wild soybean maintains a much higher level of genetic diversity than cultivated soybean and represents a treasured genetic resource (Xie et al., 2019). Wild soybean has evolved many eco types under the action of divergent evolution, such as tolerances to salt alkali, drought, barren soil (such as phosphorus or nitrogen deficiency), and so on (Sun et al., 2021). To provide an important theoretical basis for the evaluation of plant resources, it is necessary to explore the mechanism of these exceptional traits in wild soybean (Chen et al., 2019).

Nitrogen (N) is the most important factor limiting plant growth and development, especially at the seedling stage (Luca et al., 2019). The symbiotic nitrogen fixation system of leguminous plants, such as soybean, is not fully established at the seedling stage, and the seedings cannot or rarely use the nitrogen supplied by the symbiotic nitrogen fixation of rhizobia (Mourtzinis et al., 2018). Cultivating low nitrogen (LN) tolerant crop varieties can solve the problem of environmental pollution caused by the application of excessive nitrogen fertilizer in global agricultural production (Arun et al., 2016; Mourtzinis et al., 2018). Wild soybean has high levels of stress tolerance and is an important germplasm resource for improving the stress tolerance of cultivated soybean. It has been reported that, under low-nitrogen stress in the leaves of wild soybean seedlings, the concentration of carotenoids, which can be used as a photo-protective substance to inhibit the increase of reactive oxygen species (ROS) (Zhao et al., 2019). Wild soybean could maintain the balance of carbon and nitrogen metabolism by regulating the activities of nitrogen metabolism-related enzymes to improve the ability to tolerate low-nitrogen stress (Wang et al., 2019). Therefore, it is necessary to improve the LN tolerance of soybean by using appropriate wild germplasm resources.

In recent years, metabolomics and its correlation with ion concentrations had been widely used to determine responses to various abiotic stresses, including salinity, drought, and nutritional deficits (Li et al., 2017; Jiao et al., 2018). Metabolomics provides insights into the deep relationship between metabolites and changes in plant physiological conditions by combining a range of different analytical technologies and calculation methods (Yi et al., 2016). Under nutrient deficiencies, plant autophagic systems and vacuolar proteinases can degrade nitrogen-based macromolecular substances into amino acids, peptides, and urea, and then transport them to nitrogen-demanding organs (Galloway et al., 2004). Metabolomics analysis showed that wild soybean could improve its ability to tolerate nitrogen stress by increasing rates of secondary metabolism, carbohydrate metabolism and amino acid metabolism in the roots (Sun et al., 2021). During senescence of plant leaves, the catabolic processes of macromolecular compounds increase, whereas anabolic processes decrease, and the mobilization and recycling of nutrients occur from old leaves to sinks, such as young leaves, especially under conditions of nutrient deficiency, similar findings have been reported in *Arabidopsis thaliana* (Mei & Thimann, 1984).

The transfer of some nutrients from senescent tissues to other, more demanding tissues in plants is called nutrient reabsorption (Kichey et al., 2007; Elger et al., 2009). This process can improve the utilization efficiency of nutrients by plants and reduce the dependence on soil nutrients (Taylor et al., 2010; Wei et al., 2018). Nutrient reabsorption is an important mechanism for plants to improve their ability to survive under adversity. Amino acids are important metabolites and carriers of organic nitrogen between plant organs (Häusler, Ludewig & Krueger, 2014). Under stress, amino acids are subsequently recycled and allocated for the synthesis of specific proteins required under nutrient limitation (Araújo et al., 2011). Whereas during carbon or nitrogen starvation, proteins are degraded, and the complete oxidation of their amino acids produces the energy required to fuel the particular needs of stressed leaves. Furthermore, nitrogen uptake rates by roots of intact soybean plants are stimulated by malate, which moves down the phloem and accumulates in the root (López-Bucio et al., 2000). Understanding the relationship between organic acid metabolism and nitrate uptake and utilization could be an important avenue to understand the survival strategies of barren-tolerant wild soybean. The combined analysis of physiological and metabolite profiles has provided a new perspective with which to reveal the reuse of nitrogen between young and old leaves of legumes under LN conditions (Tegeder & Masclaux-Daubresse, 2018).

In the current study, to analyze the tolerance strategies used by wild soybean ecotypes adapted to LN stress, common wild soybean (GS1) and barren-tolerant wild soybean (GS2) were used as the study materials. LN stress was simulated using Hoagland’s nutrient solution containing one-quarter of the non-limiting nitrogen concentration. The photosynthetic rate, ion concentration changes, and the types, quantities, and pathways of small molecular organic metabolites in young and old leaves of the two accessions were determined and analyzed under LN stress. By comparing the differences in leaf nutrient reabsorption between the two ecotypes of wild soybean under LN stress, the mechanism of LN tolerance employed by wild soybean which evolved under divergent adaptation conditions are discussed from the perspective of nutrient reabsorption. The present study provides a quantitative standard by which to select, evaluate and utilize wild soybean germplasm accessions and provides an important scientific basis for research on plant divergent evolution.

## Materials and Methods

### Plant materials and growth conditions

The experimental materials were common wild soybean (‘Huinan 06116’, GS1) and barren-tolerant wild soybean (‘Tongyu 06311’, GS2). GS1 is from Huinan County, Tonghua City, Southeast Jilin Province. The soil here is fertile and nutrient rich. The humus layer is deep, and the organic matter content of the soil surface is high, which contains rich nitrogen, phosphorus, sulfur and other nutrients. GS2 was collected in Tongyu County, Baicheng City, western Jilin Province, where the soil is poor and associated by salinization. Three seeds were uniformly sown in each 14-cm diameter plastic pot, with each pot containing 2.5 kg of washed sand and the seeds were covered with clean sand. The pots were irrigation with water. The experimental materials were cultivated in an outdoor experimental field at Northeast Normal University, Changchun, Jilin. The pots were placed outdoors but were sheltered from the rain. The average temperatures were 18.5 ± 1.5 °C and 26 ± 2 °C during the night and day, respectively, and the relative humidity was 60 ± 5%.

### Treatments

One seedling per pot was selected based on its growth, and the other two were removed. When the seedlings had produced their third (compound) leaves, GS1 and GS2 were both randomly divided into two groups of eight pots each: control (CK) and low nitrogen (LN)-treated. CK plants of GS1 and GS2 accessions were cultivated under normal conditions, with seedlings irrigated with Seeds and seedlings were irrigated with 1 × Hoagland’s nutrient solution (Table S1). The concentrations of Ca(NO_3_)_2_·4H_2_O and KNO_3_ in 1 × Hoagland’s nutrient solution were 3.45 and 5.01 mmol L^−1^, respectively. In the LN-treated group, GS1 and GS2 seeds were placed in 1/4-strength Hoagland’s nutrient solution, where the concentrations of Ca(NO_3_)_2_·4H_2_O and KNO_3_ were 0.87 and 1.25 mmol L^−1^, respectively. The deficiency of calcium and potassium ions in the LN stress group were corrected by supplying seedlings with equivalent concentrations of CaCl_2_·2H_2_O and KCl of 2.61 and 3.75 mmol L^−1^, respectively. After 2 weeks, four replicate pots of each of the four treatments (LN/CK × GS1/GS2) were randomly used for measuring physiological parameters, ion content and the remaining four pots were used for metabolomics analyses.

### Measurements of physiological parameters

After the soybean plants were harvested, shoot height, root length, shoot dry weight (DW) and root shoot dry weight (DW) were measured for the four replicates of each treatment (Shi et al., 2015). A subsample (1 mg) of crushed dry tissue was used to measure the total nitrogen content (TN) (%) and total carbon content (TC) (%) by stable isotope mass spectroscopy (Iso Prime Elemental Analyzer, Isoprime Ltd., Tokyo, Japan), with three technical replicates measured for each replicate (Liu et al., 2020).

The leaves attached to the first and second nodes from the top of the plant were regarded as the young leaves, and the leaves attached to the first and second nodes from the bottom of the plant were regarded as old leaves for parameter determination. The gas-exchange parameters, namely net photosynthetic rate (*P*_N_), transpiration rate (*E*), stomatal conductance (*g*_s_), and substomatal CO_2_ concentration/atmospheric CO_2_ concentrations (*C*_i_/*C*_a_) of young leaves and old leaves were measured at a photosynthetic irradiation of 1,200 ± 50 µmol C^−2^ s^−1^ using LI-6400 portable photosynthesis apparatus (LI-COR, Lincoln, NE, USA) with an open system. Approximately 500 mg of fresh leaves were used to measure the concentrations of chlorophyll a (*Chl* a), chlorophyll b (*Chl* b) and carotenoid (*Car*). *Chl* a, *Chl* b and *Car* were extracted with acetone and were measured using spectrophotometer at 645, 663 and 440 nm, respectively (Holm, 1954; Gao et al., 2022) (Spec trUV-754; Shanghai Accurate Scientific Instrument Co., Shanghai, China). All measurements were performed three times for each biological replicate.

### Measurements of ion concentrations

Briefly, 0.05 g dry tissue samples were treated with 4 mL of deionized water at 100 °C for 40 min and then centrifuged at 3,000 ×g for 15 min. The supernatant was collected, and the process was repeated on the residue twice more until the pooled supernatants totaled 15 mL and were used to determine anion concentrations of Cl^−^, NO_3_^−^, H_2_PO_4_^−^, SO_4_^2−^ and oxalate (C_2_O_4_^2−^) by ion chromatography (DX-300 ion chromatographic system, AS4A-SC chromatographic column, CDM-II electrical conductivity detector; mobile phase: Na_2_CO_3_/NaHCO_3_ = 1.7/1.8 mM, Dionex, Sunnyvale, CA, USA). The concentrations of cations Mn^2+^, B^3+^, Fe^2+^, Zn^2+^, Na^+^, Mg^2+^, Ca^2+^ and K^+^ were determined using an atomic absorption spectrophotometer (Super 990F; Beijing Purkinje General Instrument Co. Ltd., Beijing, China).

### Metabolite profiling analysis

For metabolomics, metabolites were independently extracted from 100 ± 5 mg samples of the young and old leaves of accessions GS1 and GS2, and a gas chromatography–mass spectrometry (GC–MS) analysis was performed using a one-dimensional Agilent 7890 gas chromatography system coupled with a Pegasus HT time-of-flight mass spectrometer. The data was acquired and pre-processed using the manufacturer’s ChromaTOF software (versions 2.12, 2.22, 3.34; LECO, St. Joseph, MI, USA). Data analysis was performed using the SIMCA-P 13.0 software package (Umetrics, Umea, Sweden). Finally, to analyst the significance of changes in metabolite concentration compared with the corresponding control, Student’s ttest (*p* < 0.05), VIP > 1, and similarity value >700 were used to screen differential metabolites. Then, the Kyoto Encyclopedia of Genes and Genomes (KEGG) database was used to analyze metabolites and construct metabolic pathways, and the MetaboAnalyst website (https://www.metaboanalyst.ca/) was used to analyze the pathways (Xia et al., 2012).

### Data analysis

All measurements were repeated three times, and the data organized in Microsoft Excel 2007. The data values are presented as mean ± standard error (SE). The data were analyzed statistically by two-way analysis of variance (ANOVA) in SPSS (v.13.0; IBM, Armonk, NY, USA) and significant differences among treatment means were detected at *p* < 0.05 by pairwise multiple comparisons, using Duncan’s multiple range test. Sigma Plot 10.0. was used to draw the figure graphics, all of which show data points and error bars as the mean ± SE. To aid interpretation, principal component analysis (PCA) of the ionomic and metabolic were performed for both species.

## Results

### Morphological characteristic

Compared with the CK, shoot height decreased significantly 0.12-fold (*p* < 0.05) in GS1, but there was no significant change in GS2 under LN. Root length increased significantly 0.05-fold (*p* < 0.05) and 0.15-fold (*p* < 0.05) in GS1 and GS2, respectively under LN. Under LN, the shoot height of GS1 decreased more and the root length increased less than that of GS2. Shoot DW decreased significantly 0.92- (*p* < 0.01) and 0.51-fold (*p* < 0.05) in the GS1 and GS2, respectively. Root DW decreased significantly 0.36-fold (*p* < 0.01) and 0.27-fold (*p* < 0.05) in GS1 and GS2, respectively. The shoot DW and root DW decreased in both ecotypes under LN compared with CK, declined particularly in GS1 (Table S2). No significant changes in TN (%) and TC (%) in young leaves occurred in GS1 and GS2 (*p >* 0.05), whereas, in old leaves, TN (%) decreased significantly 0.35-fold and 0.13-fold (*p* < 0.05) in GS1 and GS2, respectively. Compared with CK, the TC/TN ratio increased significantly by 0.25-fold (*p* < 0.05) in GS1 and 0.17-fold (*p* < 0.01) in GS2 in old leaves under LN (Table 1). The number and weights of nodules in the seedlings of two wild soybean ecotypes did not vary significantly between CK and LN conditions. Overall, the results showed that the tolerant ecotype GS2 was less affected than GS1 under low-nitrogen stress.

**Table 1:** Carbon and nitrogen contents in young and old leaves of two wild soybean varieties under LN stress.

		GS1		GS2		Fold changes Log_2_^(LN/CK)^	
		CK	LN	CK	LN	GS1	GS2
YL	TN(%)	5.62 ± 0.16	5.13 ± 0.25	5.66 ± 0.16	6.16 ± 0.02	−0.13	0.12
	TC(%)	40.61 ± 1.19	38.63 ± 1.79	40.44 ± 1.36	43.27 ± 0.07	−0.07	0.10
	TC/TN	7.23 ± 0.03	7.54 ± 0.03	7.14 ± 0.04	7.03 ± 0.02	0.06	−0.02
OL	TN(%)	5.01 ± 0.05	3.94 ± 0.14	4.77 ± 0.07	4.38 ± 0.05	−0.35*	−0.13*
	TC(%)	40.36 ± 0.06	37.80 ± 0.85	40.26 ± 1.24	41.55 ± 0.18	−0.1	0.05
	TC/TN	8.06 ± 0.08	9.60 ± 0.14	8.43 ± 0.14	9.5 ± 0.09	0.25*	0.17**

**Note:**

Values were presented as the mean ± standard error of four biological replicates. GS1, common wild soybean; W, LN-tolerant wild soybean; CK, control treatment; LN, low nitrogen stress; YL, young leaves; OL, old leaves; C/N, Carbon/Nitrogen; one asterisk (*) indicates significance (*p* < 0.05), two asterisks (**) indicate significance (*p* < 0.01).

### Photosynthesis characters

**Figure 1:**
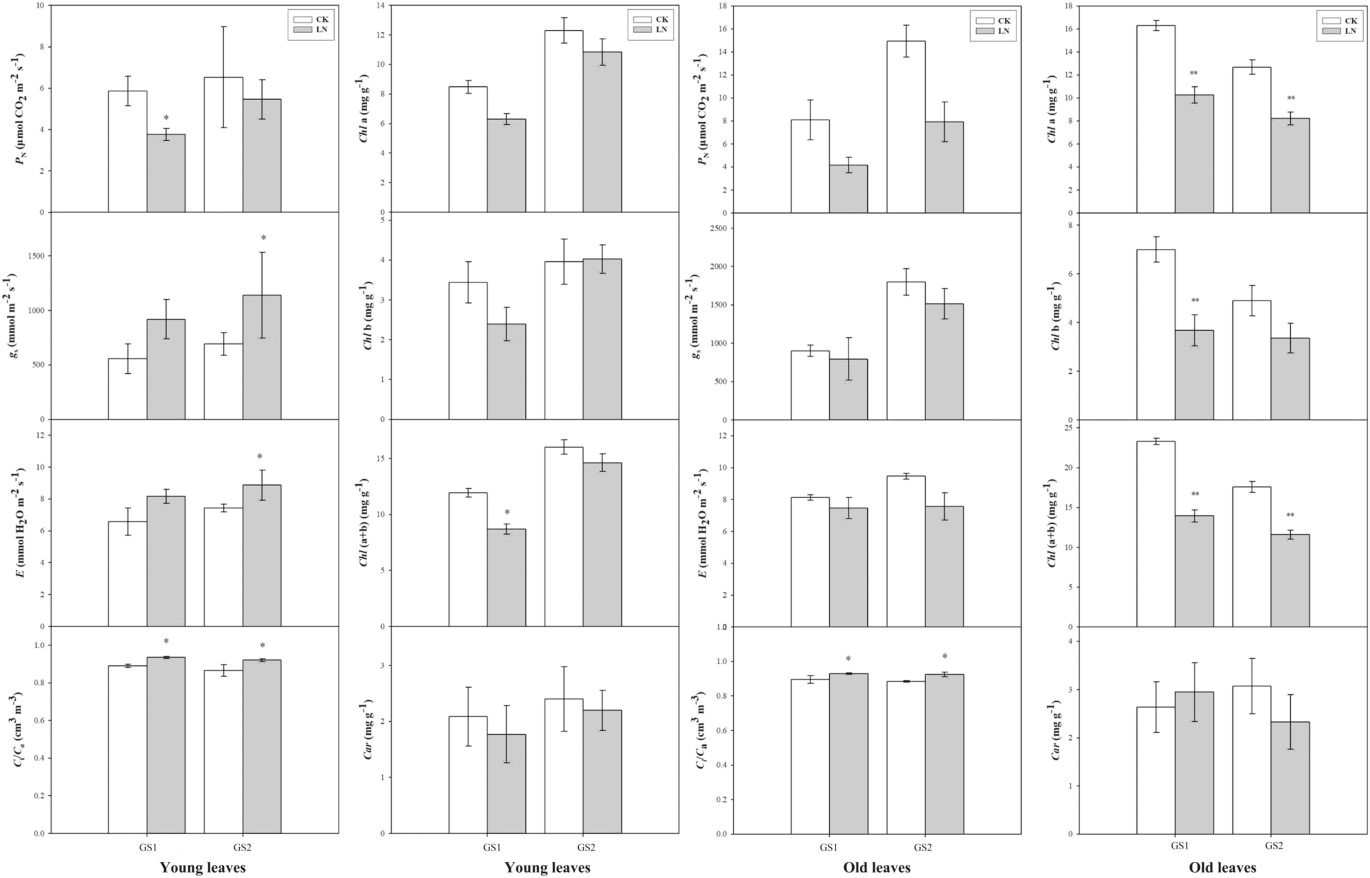
The changes in gas exchange of the two soybean ecotypes young and old leaves. *P*, net photosynthetic rate; *g*, stomatal conductance; *E*, transpiration rate; *C*/C, ratio of sub-stomatal to atmospheric CO concentrations; GS1, common soybean; GS2, barren tolerant wild soybean. CK, control treatment; LN, low-nitrogen stress; one asterisk (*) indicates significance (*p* < 0.05), two asterisks (**) indicate significance (*p* < 0.01).

Compared with the corresponding plants under CK conditions, *P*_N_ decreased significantly 0.64-fold (*p* < 0.05) and 0.26-fold (*p* > 0.05) in the young leaves of GS1 and GS2 respectively. *C*_i_/*C*_a_ increased significantly 0.07-fold (*p* < 0.05) and 0.09-fold (*p* < 0.05) in the young leaves of GS1 and GS2, respectively, and increased 0.05-fold (*p* < 0.05) and 0.07-fold (*p* < 0.05) in the old leaves of GS1 and GS2, respectively. *Chl(a+b)* decreased significantly in the old leaves of GS1 and GS2, especially in GS1 significantly decreased (*p* < 0.05) (Fig.1).

### Response of ion concentrations

**Figure 2:**
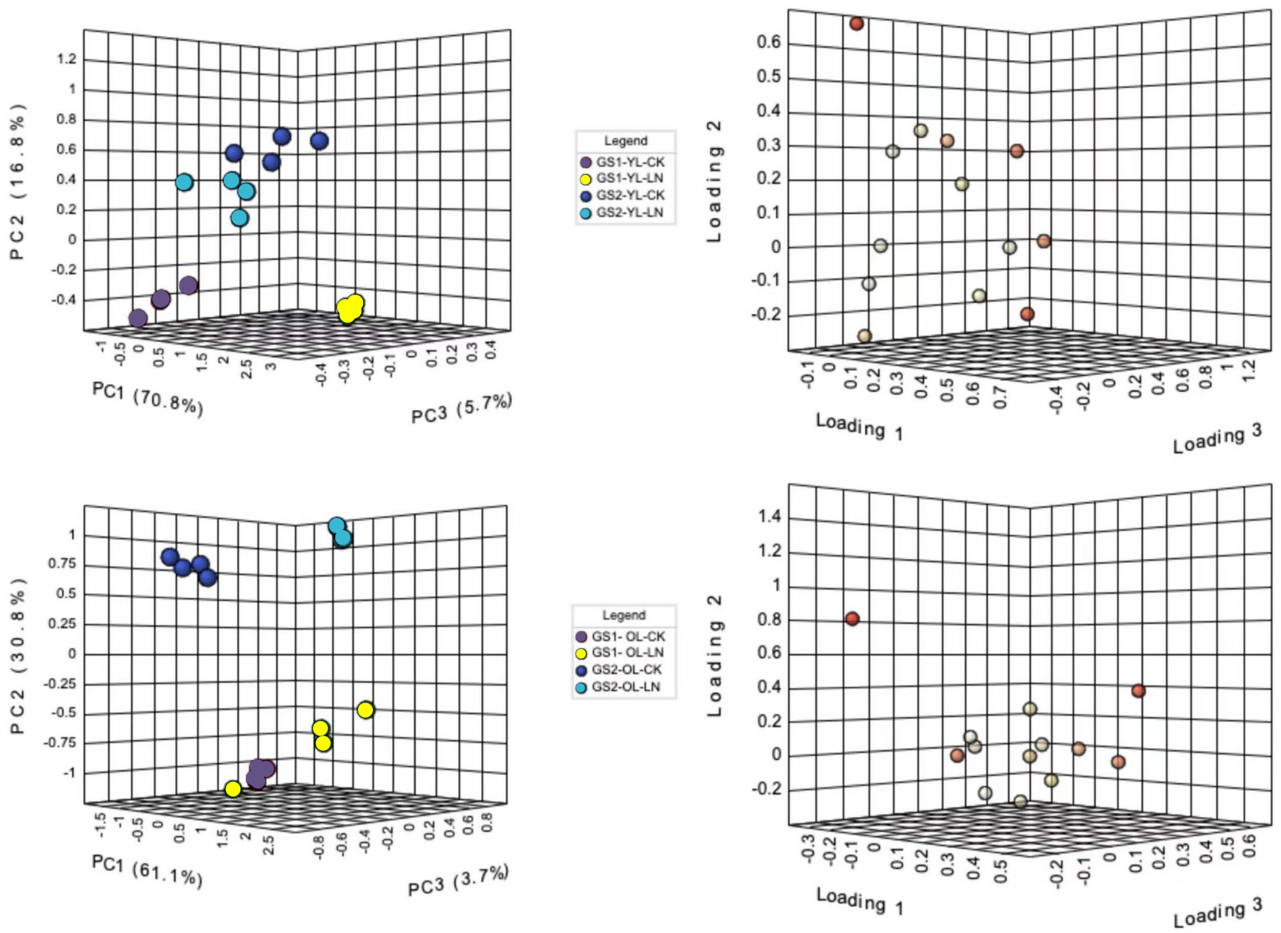
PCA of ionomic proûles and loading plots of ionomics in young and old leaves. (A) PCA of young leaves; (B) loading plot of young leaves; (C) PCA of old leaves; (D) loading plot of old leaves; GS1, common soybean; GS2, barren tolerant wild soybean; CK, control treatment; LN, low-nitrogen stress.

Principal component analysis (PCA) was used to differentiate the ion concentrations in the young and old leaves of the two ecotypes of wild soybeans (Fig. 2). The ion concentrations of young leaves in the CK and LN treatments were clearly separated by the first component (PC1), representing 70.9% of the total variation, and NO_3_^−^, SO_4_^2−^, SO_4_^2−^, Cl^−^, B^3+^ and Fe^2+^ were the major contributors (Figs. 2A and 2B; Table S3). PC2 represented 16.9% of the variation between the different ecotypes, of which NO_3_^−^, Mn^2+^, H_2_PO_4_^−^ and Na^+^ were major contributors (Fig. 2A and 2B; Table S3). The CK and LN ion content in old leaves were clearly separated by PC1, representing 58.9% of the total variation, and NO_3_^−^, Mg^2+^, Ca^2+^, Cl^−^, B^3+^, and Fe^2+^ were the major contributors (Figs. 2C and 2D; Table S3). PC2 presented a 13.2% variation between GS1 and GS2, and H_2_PO_4_^−^, SO_4_^2−^, C_2_O_4_^2−^, Mn^2+^, and Na^+^ were major contributors (Figs. 2C and 2D; Table S3).

Under LN stress, NO_3_^−^ decreased significantly 0.69-fold and 0.50-fold (*p* < 0.01) and SO_4_^2−^ decreased significantly 0.15-fold and 0.29-fold (*p* < 0.05) in the young leaves of GS1 and GS2, respectively. Na^+^ and Ca^2+^ concentrations increased significantly 0.34-fold and 0.16-fold (*p* < 0.05) in the young leaves of GS2. and there was particularly significant enrichment of Mn^2+^ and Zn^2+^ concentrations in the young leaves of GS2. Under LN. The levels of B^3+^, Fe^2+^, H_2_PO_4_^−^and C_2_O_4_^2−^ increased significantly in GS1 young leaves (*p* < 0.05) and highly significantly in GS2 young leaves (*p* < 0.01). NO_3_^−^ concentrations decreased significantly2.10-fold and 1.77-fold (*p* < 0.01) in the old leaves of GS1 and GS2, respectively, whereas SO_4_^2−^ increased 0.37-fold (*p* < 0.01) in GS1 but decreased 0.98-fold (*p* < 0.01) in the old leaves of GS2. The concentrations of Cl^−^, H_2_PO_4_^−^, Mn^2+^, B^3+^, Fe^2+^, and Zn^2+^ increased in the old leaves of GS2 (Table 2).

**Table 2:** Ion contents in young and old leaves of two wild soybean ecotypes under LN stress values were presented as the mean ± standard error of four biological replicates.

		GS1		GS2		Fold changes Log_2_^(LN/CK)^	
		CK	LN	CK	LN	GS1	GS2
Young leaves	Cl^−^	2.39 ± 0.11	4.47 ± 0.07	2.27 ± 0.17	4.93 ± 0.20	0.90	1.12**
	NO_3_^−^	2.26 ± 0.11	1.40 ± 0.01	3.5 ± 0.05	2.48 ± 0.04	−0.69**	−0.50**
	H_2_PO_4_^−^	18.57 ± 0.0.26	31.96 ± 0.97	19.72 ±	23.35 ± 0.31	0.78*	0.24**
	SO_4_^2−^	26.11 ± 0.20	23.59 ± 0.70	31.5 ± 0.94	25.85 ± 2.23	−0.15*	−0.29**
	C_2_O_4_^2−^	1.03 ± 0.06	1.72 ± 0.02	1.58 ± 0.04	1.69 ± 0.08	0.74*	0.10**
	Mn^2+^	0.09 ± 0.00	0.13 ± 0.00	0.11 ± 0.00	0.20 ± 0.01	0.59*	0.84**
	B^3+^	0.35 ± 0.01	1.62 ± 0.00	0.53 ± 0.01	1.87 ± 0.07	2.22*	1.81**
	Fe^2+^	0.05 ± 0.00	0.12 ± 0.00	0.06 ± 0.00	0.13 ± 0.01	1.17*	1.07**
	Zn^2+^	0.14 ± 0.01	0.24 ± 0.00	0.17 ± 0.01	0.35 ± 0.01	0.78	1.06**
	Na^+^	2.15 ± 0.15	2.05 ± 0.05	1.64 ± 0.08	2.08 ± 0.15	−0.07	0.34*
	Mg^2+^	28.30 ± 0.79	26.48 ± 0.13	30.14 ± 0.28	29.57 ± 0.92	−0.10	−0.03
	Ca^2+^	14.61 ± 0.41	14.00 ± 0.05	13.44 ± 0.07	14.97 ± 0.41	−0.06	0.16*
	K^+^	191.66 ± 5.18	169.57 ± 0.30	167.2 ± 1.48	174.03 ± 5.27	−0.18*	0.06
Old leaves	Cl^−^	2.49 ± 0.18	7.77 ± 0.24	1.83 ± 0.08	7.92 ± 0.21	1.64	2.12**
	NO_3_^−^	3.96 ± 0.04	0.92 ± 0.04	4.24 ± 0.05	1.24 ± 0.04	−2.10**	−1.77**
	H_2_PO_4_^−^	20.58 ± 0.05	46.04 ± 0.66	16.1 ± 0.45	25.01 ± 0.18	1.16**	0.64**
	SO_4_^2−^	2.16 ± 0.12	2.79 ± 0.11	55.39 ± 1.34	28.07 ± 0.30	0.37**	−0.98**
	C_2_O_4_^2−^	1.33 ± 0.00	1.34 ± 0.04	1.26 ± 0.08	1.14 ± 0.05	0.01	−0.13
	Mn^2+^	0.12 ± 0.02	0.16 ± 0.04	0.22 ± 0.03	0.3 ± 0.01	0.46	0.43*
	B^3+^	1.09 ± 0.03	4.86 ± 0.05	1.18 ± 0.13	4.94 ± 0.16	2.16**	2.07**
	Fe^2+^	0.04 ± 0.00	0.09 ± 0.00	0.06 ± 0.01	0.09 ± 0.01	0.98**	0.68*
	Zn^2+^	0.09 ± 0.00	0.35 ± 0.00	0.11 ± 0.01	0.29 ± 0.01	1.96	1.35**
	Na^+^	1.01 ± 0.04	1.82 ± 0.06	1.76 ± 0.11	2.76 ± 0.08	0.85**	0.65**
	Mg^2+^	46.40 ± 0.01	33.28 ± 0.06	60.62 ± 7.75	42.48 ± 1.55	−0.48**	−0.51
	Ca^2+^	48.96 ± 0.14	31.32 ± 0.07	58.76 ± 7.66	38.89 ± 0.69	−0.64**	−0.60*
	K^+^	163.44 ± 0.07	172.59 ± 0.70	166.64 ± 17.11	141.27 ± 3.60	0.08**	−0.24

**Note:**

GS1, common soybean; GS2, barren tolerant wild soybean; CK, control treatment; LN, low-N stress. * and ** indicate signiûcant (*p* < 0.05) and highly signiûcant (*p* < 0.01) diûerences, respectively.

### Metabolomics response in young leaves

**Figure 3:**
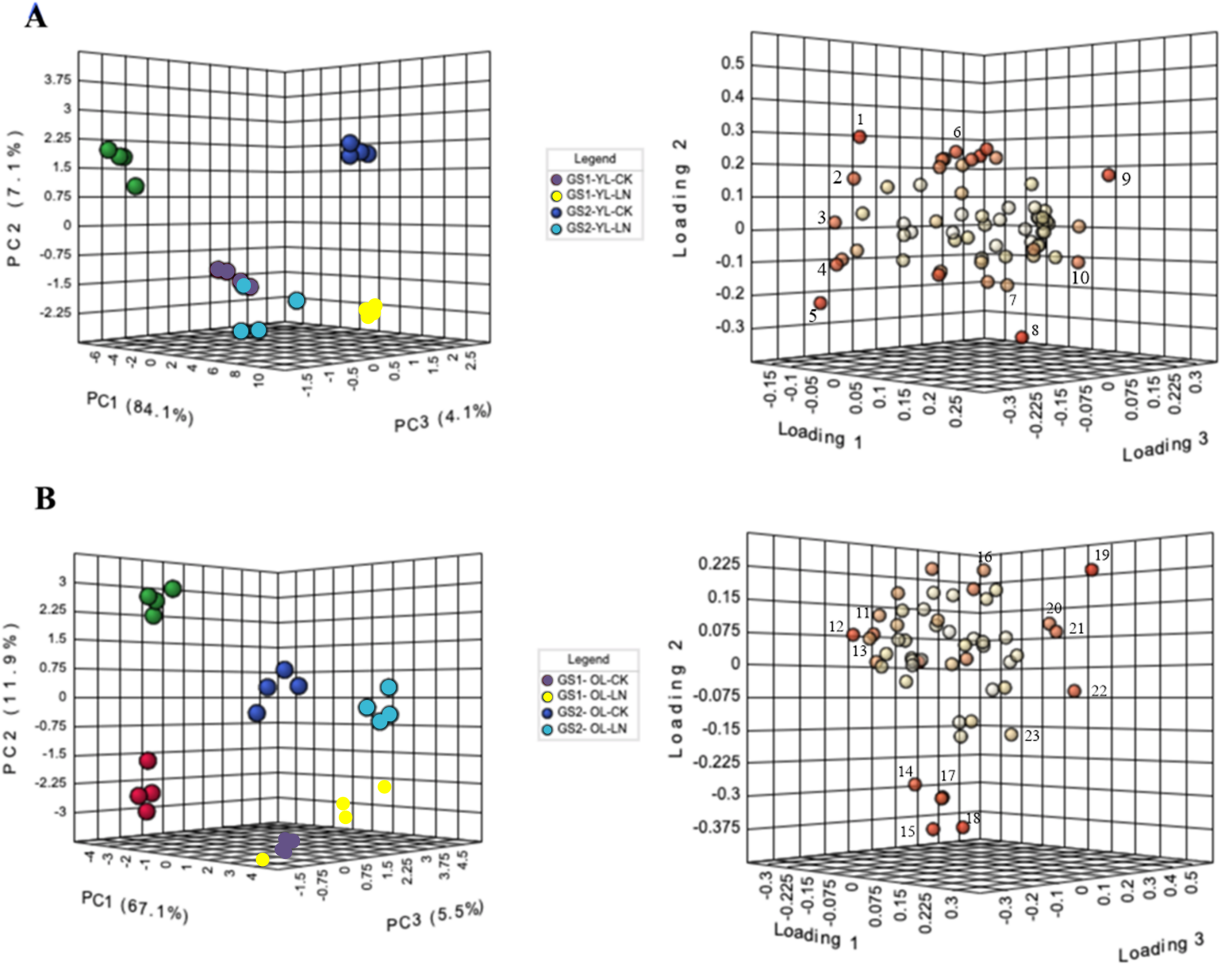
PCA of metabolic profiles and loading plots of metabolites in young and old leaves. (A) PCA of young leaves; (B) PCA of old leaves; (C) loading plot of young leaves; (D) loading plot of old leaves. GS1, common soybean; GS2, barren tolerant wild soybean; CK, control treatment; LN, low-nitrogen stress. 1, oxalic acid; 2, glucoheptonic acid; 3, threitol; 4, lactulose; 5, 3-aminoisobutyric acid; 6, 2-hydroxypyridine; 7, maleic acid; 8, 3-hydroxypropionic acid; 9, alanine; 10, palmitic acid, 11, alpha-linolenic acid; 12, L-serine; 13, Glucose-6-phosphate; 14, Vanillin; 15, maleic acid; 16, neohesperidin; 17, L-alanine; 18, L-aspartic acid; 19, benzoic acid; 20, L-proline; 21, threonine; 22, D-arabitol; 23, glyceric acid.

In PCA, PC1 explained 84% of the variance, in metabolite concentratins changes between the young leaves of GS1 and GS2 (Fig. 3). The main metabolites contributing to PC1 were alanine, 3-aminoisobutyric acid, lactulose, and threitol. The CK and LN-stressed young leaves in GS1 and GS2 were clearly distinguished by PC2, which represented 7.1% of the total variation, with the main factors contributing to PC2 were 3-hydroxypropionic acid, 2-hydroxypyridine, oxalic acid, and maleic acid (Fig. 3A, Table 3 and Table S4). PC3 explained 4.1% of the variance and the main contributors were glucoheptonic acid, threitol, maleic acid, and palmitic acid (Fig. 3A, Table 3 and Table S4). The levels of 63 metabolites changed significantly under LN stress relative to CK in the young leaves of both ecotypes (Table 3).

**Table 3:** Changes of LN stress on metabolite content in young leaves of two wild soybean ecotypes.

	Metabolite name	Relative concentration				Fold changes Log2(LN/CK)	
		GS1		GS2			
		CK	LN	CK	LN	GS1	GS2
Amino acids	Valine	0.62 ± 0.04	0.54 ± 0.05	8.16 ± 0.95	7.05 ± 0.29	−0.19**	−0.21
	Isoleucine	0.39 ± 0.01	0.11 ± 0.00	1.49 ± 0.28	2.76 ± 0.20	−1.83**	0.89*
	Aspartic acid	5.30 ± 0.06	9.35 ± 0.01	7.60 ± 0.51	11.39 ± 0.34	0.82**	0.58**
	Asparagine	2.58 ± 0.67	0.60 ± 0.76	0.11 ± 0.05	0.09 ± 0.01	−2.09**	−0.38
	L-Homoserine	0.22 ± 0.00	0.08 ± 0.00	0.18 ± 0.06	0.99 ± 0.03	−1.56	2.45**
	L-Allothreonine	1.77 ± 0.03	1.82 ± 0.04	2.44 ± 0.15	0.81 ± 0.09	0.04	−1.58**
	Serine	0.19 ± 0.13	0.16 ± 0.25	0.90 ± 0.14	0.68 ± 0.07	−0.27	0.41
	Proline	0.27 ± 0.00	0.21 ± 0.02	0.22 ± 0.02	0.50 ± 0.03	−0.40*	1.21**
	Alanine	0.06 ± 0.04	0.07 ± 0.03	5.05 ± 0.36	24.78 ± 0.75	0.35	2.29**
	Glycine	28.26 ± 0.02	50.61 ± 0.04	6.69 ± 0.57	4.58 ± 0.27	0.84*	−0.54*
	Phenylalanine	11.31 ± 0.07	10.73 ± 0.02	1.89 ± 0.28	0.69 ± 0.13	−0.08	−1.45*
	4-Aminobutyric acid	0.52 ± 0.01	0.32 ± 0.03	2.37 ± 0.12	2.73 ± 0.28	−0.70*	0.21*
	Ethanolamine	55.44 ± 0.02	86.12 ± 0.04	15.31 ± 0.55	8.50 ± 0.35	0.64*	−0.85**
	D-Mannosamine	11.32 ± 0.00	7.19 ± 0.01	0.06 ± 0.05	0.08 ± 0.01	−0.65	0.25
	Sphingosine	1.76 ± 0.30	2.34 ± 0.34	3.08 ± 0.32	2.85 ± 0.54	0.41	−0.11
	3-Aminoisobutyric acid	0.41 ± 0.02	0.23 ± 0.03	0.24 ± 0.02	0.25 ± 0.02	−0.85	0.08
	5-Aminovaleric acid	4.59 ± 0.14	0.80 ± 0.09	0.29 ± 0.07	0.62 ± 0.08	−2.53	1.07*
	2-Hydroxypyridine	0.07 ± 0.00	0.01 ± 0.00	7.20 ± 0.62	7.58 ± 0.25	−2.32	0.07
Sugars and polyols	Sucrose	0.08 ± 0.07	0.07 ± 0.00	1.30 ± 0.17	5.28 ± 0.44	−0.10	2.02**
	Maltose	0.33 ± 0.01	0.28 ± 0.02	0.30 ± 0.04	1.48 ± 0.14	−0.21	2.33**
	Maltotriose	0.93 ± 0.02	0.96 ± 0.03	0.09 ± 0.01	0.05 ± 0.03	0.04	−0.73
	Melezitose	28.10 ± 0.38	20.39 ± 0.02	0.06 ± 0.01	1.29 ± 0.25	−0.46	4.55**
	Threitol	2.57 ± 0.20	1.72 ± 0.08	0.29 ± 0.01	1.17 ± 0.16	−0.58	2.04**
	Galactinol	0.71 ± 0.01	0.52 ± 0.02	0.92 ± 0.09	1.03 ± 0.03	−0.47	0.16
	Glycerol	1.91 ± 0.28	1.56 ± 0.27	8.33 ± 0.27	6.66 ± 0.82	−0.29	−0.32
	Myo-inositol	0.31 ± 0.02	0.22 ± 0.03	43.71 ± 0.55	2.15 ± 0.33	−0.48	−4.35**
	L-Threose	54.13 ± 0.01	60.66 ± 0.16	0.08 ± 0.01	0.02 ± 0.01	0.16	−1.67**
	Xylose	0.09 ± 0.02	0.11 ± 0.01	1.32 ± 0.18	1.37 ± 0.25	0.29	0.05
	Cellobiose	0.98 ± 0.06	0.60 ± 0.02	0.04 ± 0.01	0.31 ± 0.04	−0.72	2.83**
	Lactulose	1.95 ± 0.12	0.05 ± 0.03	0.33 ± 0.04	1.71 ± 0.47	− 5.28	2.39*
	D-Arabitol	0.25 ± 0.13	0.97 ± 0.06	2.28 ± 0.36	1.18 ± 0.09	1.96	0.95*
	Glucose-6-Phosphate	306.86 ± 0.34	216.33 ± 0.17	0.03 ± 0.00	0.03 ± 0.00	−0.50*	0.33
	Fructose 2,6-Biphosphate	0.08 ± 0.00	0.28 ± 0.01	0.15 ± 0.02	0.07 ± 0.02	1.84**	−1.12*
	Maltose	1.50 ± 0.22	2.40 ± 0.25	0.25 ± 0.02	0.61 ± 0.14	0.68	1.30*
Organic acid	Glycolic acid	8.09 ± 0.33	4.01 ± 0.07	0.12 ± 0.00	0.74 ± 0.19	−1.01	2.67*
	Lactic acid	0.15 ± 0.02	0.16 ± 0.04	2.97 ± 0.38	3.47 ± 0.14	0.09	0.22
	Methylmalonic acid	0.55 ± 0.08	0.06 ± 0.01	0.12 ± 0.00	0.08 ± 0.01	−3.13	−0.57*
	Mucic acid	0.20 ± 0.02	0.13 ± 0.02	1.45 ± 0.16	1.84 ± 0.09	−0.64	0.34
	D-Glyceric acid	0.33 ± 0.55	0.43 ± 0.09	2.52 ± 0.25	1.81 ± 0.52	0.36	−0.47
	Pyruvic acid	0.20 ± 0.55	0.13 ± 0.30	1.93 ± 0.08	4.41 ± 0.50	−0.64*	1.19**
	Succinic acid	0.25 ± 0.02	0.32 ± 0.02	6.97 ± 0.69	6.53 ± 0.48	0.37*	−0.09
	Fumaric acid	4.50 ± 0.25	2.40 ± 0.16	3.08 ± 0.04	25.36 ± 1.99	−0.91*	3.04**
	Citramalic acid	7.06 ± 0.3	1.09 ± 0.02	0.45 ± 0.02	0.18 ± 0.04	−2.70**	−1.31**
	Oxalic acid	0.97 ± 1.79	0.78 ± 0.03	0.24 ± 0.02	0.17 ± 0.03	−0.31	−0.49
	Itaconic acid	2.90 ± 0.03	3.91 ± 1.79	3.07 ± 0.35	2.33 ± 0.34	0.43	−0.40
	Malonic acid	4.47 ± 0.08	5.48 ± 0.51	1.12 ± 0.06	9.71 ± 0.55	0.30	3.12**
	Maleic acid	0.08 ± 0.03	0.18 ± 0.03	6.99 ± 0.39	9.24 ± 0.90	1.11*	0.40
	Gluconic acid	0.56 ± 0.07	0.78 ± 0.09	0.07 ± 0.00	0.08 ± 0.02	0.49**	0.16
	Nicotinic acid	4.95 ± 0.58	10.38 ± 0.40	0.69 ± 0.04	1.25 ± 0.15	1.07**	0.85*
	Threonic acid	2.23 ± 0.83	0.72 ± 0.00	1.44 ± 0.21	0.81 ± 0.14	−1.63*	−0.84
	Galactonic acid	0.83 ± 0.00	0.51 ± 0.10	1.01 ± 0.04	1.12 ± 0.08	−0.70**	0.15
	Glucoheptonic acid	16.23 ± 0.31	8.81 ± 0.41	0.22 ± 0.03	0.56 ± 0.02	−0.88	1.37**
	3-Hydroxypropionic acid	0.04 ± 0.00	0.04 ± 0.00	0.19 ± 0.01	0.14 ± 0.01	−0.14	−0.40
	Shikimic acid	0.01 ± 0.00	0.00 ± 0.00	3.90 ± 0.21	1.92 ± 0.49	−1.87	−1.02*
	Stearic acid	0.24 ± 0.01	0.11 ± 0.03	0.24 ± 0.02	0.30 ± 0.03	−1.18*	0.35
	Palmitic acid	42.32 ± 1.77	40.01 ± 1.53	0.32 ± 0.06	0.44 ± 0.14	−0.08	0.48
	Linolenic acid	0.17 ± 0.05	0.14 ± 0.02	1.53 ± 0.12	0.18 ± 0.02	−0.29	−3.06**
	Linoleic acid	4.13 ± 0.09	2.72 ± 0.13	1.82 ± 0.40	2.34 ± 0.19	−0.60**	0.37
	Ferulic acid	13.46 ± 0.03	36.23 ± 0.13	1.58 ± 0.26	0.90 ± 0.12	1.43**	−0.81
	Salicylic acid	0.13 ± 0.34	0.18 ± 0.13	0.39 ± 0.03	0.05 ± 0.02	0.52	−2.94**
	Caffeic acid	1.30 ± 0.00	0.36 ± 0.00	0.02 ± 0.00	0.01 ± 0.00	−1.86*	−0.96
Flavonoids	Neohesperidin	11.03 ± 0.05	7.74 ± 0.21	0.15 ± 0.02	1.02 ± 0.84	−0.51*	2.78
	Prunin	8.47 ± 1.06	13.32 ± 0.36	0.09 ± 0.01	0.23 ± 0.02	0.65**	1.37**

**Note:**

Values were presented as the mean ± standard error of four biological replicates. GS1, common soybean; GS2, barren tolerant wild soybean; CK, control treatment; LN, low2N stress. * and ** indicate signiûcant (*p* < 0.05) and highly signiûcant (*p* < 0.01) diûerences, respectively.

Under LN stress, the concentrations of amino acids increased significantly relative to those of aspartic acid and alanine in young leaves of both ecotypes, whereas the relative concentration of asparagine and valine decreased significantly in the young leaves of GS1 but did not change significantly in those of GS2. The relative concentration of isoleucine, L-homoserine and proline increased significantly 0.89-fold (*p* < 0.05), 2.45-fold (*p* < 0.01), and 1.21-fold (*p* < 0.01), respectively, in the young leaves of GS2 whereas they decreased in those of GS1. The concentration of 4-aminobutyric acid (GABA) content decreased significantly by 0.70-fold (*p* < 0.05) in the young leaves of GS1 but significantly increased 0.21-fold (*p* < 0.05) in GS2.

**Figure 4:**
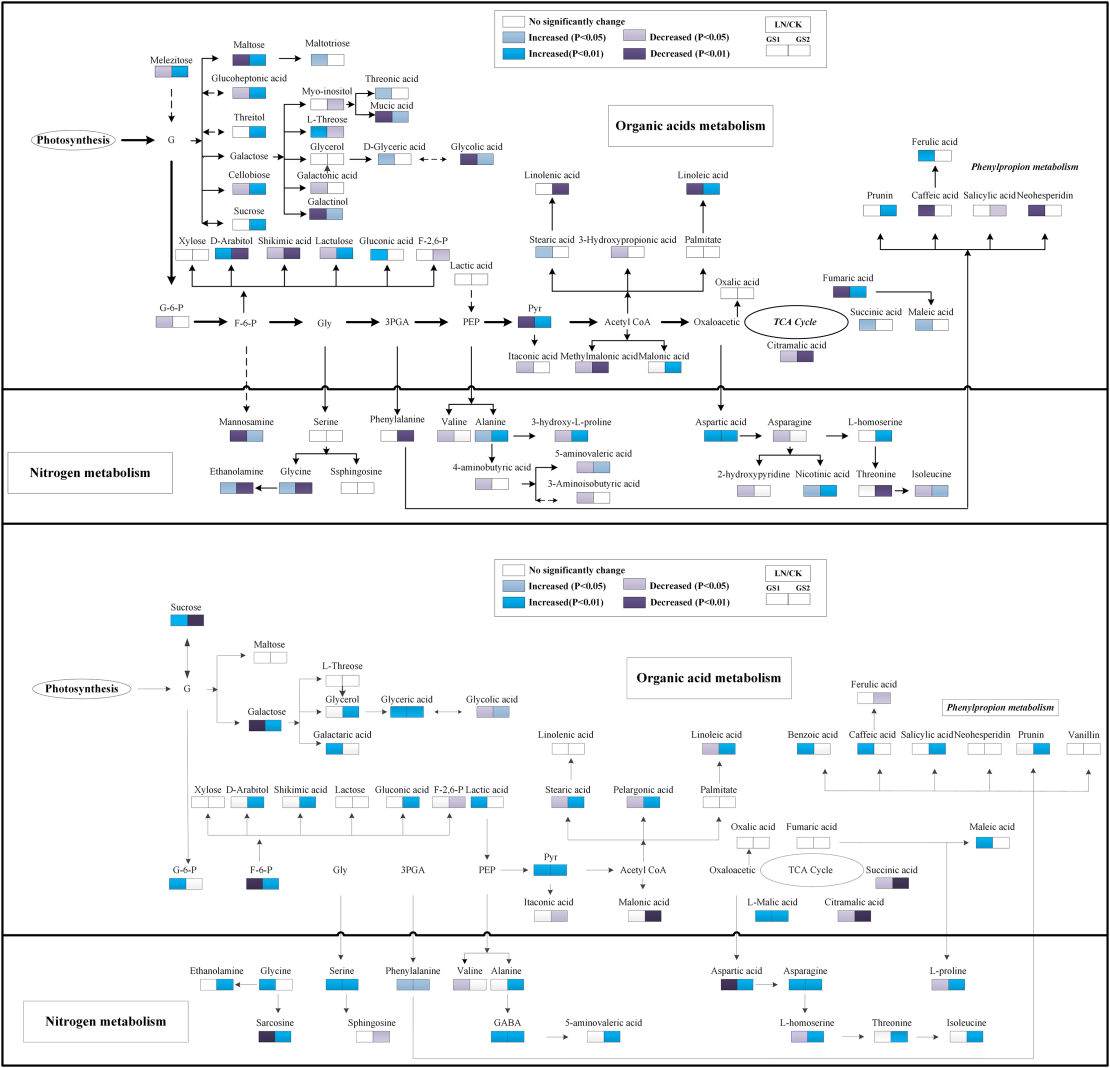
Changes in the metabolic pathways of young and old leaves in the two soybean ecotypes. Suggested changes in the metabolic network in soybean seedlings under LN-stress conditions based on a partial least square-discriminant analysis (PLS-DA). (A) Pathway of young leaves; (B) pathway of old leaves. GS1, common soybean; GS2, barren tolerant wild soybean; CK, control treatment; LN, low-nitrogen stress.

Regarding organic acids metabolites, the relative concentration of pyruvic acid and fumaric acid increased significantly under LN in the young leaves of GS2 but decreased significantly in the young leaves of GS1 compared with the CK. The relative concentration of succinic acid increased significantly 0.37-fold (*p* < 0.05) in GS1, but no significant change was observed in the young leaves of GS2 under LN stress. The relative concentration of citramalic acid decreased significantly 2.70- and 1.31-fold (*p* < 0.01) under LN in GS1 and GS2, respectively. The relative concentration of galactonic acid, threonic acid, and caffeic acid decreased significantly 0.70-fold (*p* < 0.01), 1.63-fold (*p* < 0.05), and 1.86 -fold (*p* < 0.05), respectively, under LN in young leaves of GS1, but no significant changes were observed in GS2. Ferulic acid increased significantly 1.43-fold (*p* < 0.01) in the young leaves of GS1, but no significant changes were observed in the young leaves of GS2. The relative concentration of fatty acids metabolites, including stearic acid and linoleic acid, decreased significantly by 1.18-fold (*p* < 0.05) and 0.60-fold (*p* < 0.01) in GS1 under LN, but no significant changes were observed in GS2 (Fig. 4).

### Metabolomics response in old leaves

In PCA, PC1 explained 67.1% of the variance in the old leaves between the LN and CK groups. The main metabolites that contributed to PC1 were L-serine, alpha-linolenic acid, D-arabitol, threonine, and L-proline. The old leaves of GS1 and GS2 were clearly distinguished by PC2, which represented 11.9% of the total variation, with vanillin, neohesperidin, L-aspartic acid, maleic acid, and L-alanine being the main factors in PC2. Four experimental groups were well distinguished by PC3, which represented 5.5% of the variation (Fig. 3B, Table 4 and Table S5), with vanillin, benzoic acid, glyceric acid, glucose-6-phosphate, and D-arabitol being the main contributor to PC3. The levels of 53 metabolites changed significantly under LN stress relative to CK in the old leaves of both ecotypes, including 17 N-containing compounds and 20 organic acids (Table 4).

**Table 4:** Changes of LN stress on metabolite content in old leaves of two wild soybean ecotypes.

	Metabolite name	Relative concentration				Fold changes Log2(LN/CK)	
		GS1		GS2			
		CK	LN	CK	LN	GS1	GS2
Amino acids	Proline	0.11 ± 0.01	0.81 ± 0.18	1.47 ± 0.27	0.66 ± 0.08	−1.17**	2.85**
	Glycine	1.07 ± 0.15	1.27 ± 0.30	0.08 ± 0.02	0.04 ± 0.01	−0.74*	0.25
	Sarcosine	0.81 ± 0.15	2.97 ± 0.63	0.20 ± 0.04	0.14 ± 0.00	−0.58*	1.88*
	L-Homoserine	1.04 ± 0.21	2.42 ± 0.26	0.19 ± 0.01	0.15 ± 0.00	−0.34**	1.22**
	L-Aspartic acid	0.07 ± 0.04	0.33 ± 0.09	0.21 ± 0.01	0.19 ± 0.01	−0.15*	2.28*
	L-Alanine	0.09 ± 0.01	0.26 ± 0.03	0.02 ± 0.01	0.03 ± 0.00	0.05	1.57**
	Asparagine	0.06 ± 0.01	0.22 ± 0.05	0.10 ± 0.01	0.11 ± 0.01	0.21*	1.95*
	L-Valine	37.13 ± 3.86	47.46 ± 5.60	2.11 ± 0.32	2.43 ± 0.95	0.20	0.35
	Cycloleucine	30.89 ± 5.31	44.38 ± 8.78	0.50 ± 0.17	0.64 ± 0.33	0.33	0.52
	Isoleucine	0.38 ± 0.01	1.33 ± 0.25	0.22 ± 0.05	0.30 ± 0.25	0.44	1.79**
	Beta-Alanine	0.13 ± 0.04	0.07 ± 0.02	0.06 ± 0.02	0.08 ± 0.05	0.55	−1.02
	L-Phenylalanine	0.08 ± 0.03	0.17 ± 0.02	0.15 ± 0.02	0.23 ± 0.06	0.62*	1.01*
	L-Serine	0.01 ± 0.00	0.06 ± 0.01	8.47 ± 2.47	15.09 ± 4.34	0.83*	2.32*
	Threonine	0.02 ± 0.00	0.03 ± 0.01	0.05 ± 0.01	0.10 ± 0.06	0.89	0.97*
	Sphingosine	6.28 ± 2.67	0.42 ± 0.39	0.56 ± 0.49	0.64 ± 0.45	0.18	−3.92**
	4-Aminobutyric acid	0.10 ± 0.02	0.19 ± 0.02	0.34 ± 0.03	0.41 ± 0.03	0.27	0.92*
	Ethanolamine	30.85 ± 6.18	61.33 ± 6.31	0.57 ± 0.28	1.16 ± 1.26	1.01	0.99*
	5-Aminopentanoic acid	0.73 ± 0.30	0.03 ± 0.02	0.00 ± 0.00	0.01 ± 0.01	2.63	−4.75**
Sugar and polyols	D-Galactose	0.05 ± 0.01	0.08 ± 0.01	0.03 ± 0.02	0.00 ± 0.00	−2.58*	0.53
	Lactose	0.21 ± 0.15	0.03 ± 0.02	0.08 ± 0.01	0.04 ± 0.03	−0.94	−2.82
	Gluconic acid	0.10 ± 0.16	0.34 ± 0.08	0.02 ± 0.01	0.01 ± 0.01	−0.68	1.83*
	L-Threose	4.15 ± 0.23	4.96 ± 0.75	0.01 ± 0.00	0.01 ± 0.00	0.03	0.26
	Maltose	0.12 ± 0.01	0.22 ± 0.05	0.25 ± 0.05	0.28 ± 0.19	0.13	0.91
	Xylose	6.36 ± 0.17	10.71 ± 2.81	0.05 ± 0.02	0.06 ± 0.05	0.26	0.75
	Sucrose	30.81 ± 9.34	2.64 ± 0.02	0.93 ± 0.32	1.50 ± 0.43	0.69	−3.54*
	Glycerol	0.10 ± 0.01	0.20 ± 0.00	0.02 ± 0.00	0.03 ± 0.02	0.89	0.95**
	D-Arabitol	0.12 ± 0.01	0.57 ± 0.10	0.48 ± 0.08	3.02 ± 2.41	2.64	2.23**
Organic acids	Malonic acid	0.94 ± 0.25	0.46 ± 0.17	0.90 ± 0.31	0.76 ± 0.15	−0.23	−1.04*
	Palmitic acid	0.27 ± 0.02	0.82 ± 0.26	0.04 ± 0.01	0.02 ± 0.00	−1.20*	1.59*
	Stearic acid	1.92 ± 0.10	3.50 ± 0.53	0.24 ± 0.01	0.19 ± 0.01	−0.33**	0.87*
	Pelargonic acid	0.02 ± 0.00	0.02 ± 0.00	0.01 ± 0.00	0.01 ± 0.00	−0.22**	0.27*
	Linoleic acid	8.30 ± 2.49	22.03 ± 0.87	0.00 ± 0.00	0.00 ± 0.00	−0.15**	1.41**
	Linolenic acid	3.19 ± 0.40	2.49 ± 0.33	20.27 ± 5.49	21.56 ± 6.58	0.09	−0.36
	Fructose-6−biphosphate	0.06 ± 0.00	0.15 ± 0.01	0.07 ± 0.02	0.04 ± 0.00	−0.72*	1.25**
	Pyruvic acid	0.34 ± 0.08	0.85 ± 0.11	0.02 ± 0.00	0.03 ± 0.00	0.94*	1.31*
	Glucose 6-phosphate	0.95 ± 0.15	1.55 ± 0.22	0.02 ± 0.02	0.09 ± 0.05	1.88*	0.70
	Fumaric acid	0.06 ± 0.02	0.07 ± 0.01	0.08 ± 0.00	0.06 ± 0.03	−0.52	0.11
	L-Malic acid	3.39 ± 0.57	5.29 ± 1.30	0.15 ± 0.00	0.21 ± 0.00	0.45*	0.64*
	Succinic acid	6.33 ± 1.30	10.06 ± 0.74	1.07 ± 0.29	2.24 ± 0.43	1.07*	0.67*
	Itaconic acid	0.66 ± 0.18	0.09 ± 0.02	0.08 ± 0.01	0.08 ± 0.02	−0.05	−2.91**
	Lactic acid	0.69 ± 0.09	0.93 ± 0.17	0.07 ± 0.00	0.08 ± 0.00	0.09	0.44*
	Maleic acid	1.73 ± 0.60	9.20 ± 7.08	1.73 ± 0.55	2.61 ± 0.38	0.59	2.41*
	Caffeic acid	6.48 ± 0.68	5.12 ± 0.54	0.03 ± 0.01	0.05 ± 0.00	0.67	−0.34
	Glycolic acid	0.28 ± 0.05	0.12 ± 0.04	0.12 ± 0.03	0.28 ± 0.06	1.25	−1.22
	Galactaric acid	1.05 ± 0.70	1.42 ± 0.27	0.62 ± 0.22	1.49 ± 0.55	1.26*	0.44*
	Benzoic acid	0.03 ± 0.00	0.02 ± 0.00	0.01 ± 0.01	0.14 ± 0.07	3.37*	−0.34
	Glyceric acid	9.87 ± 0.91	15.71 ± 2.56	0.00 ± 0.00	0.00 ± 0.00	4.19*	0.67*
	Shikimic acid	0.08 ± 0.00	0.15 ± 0.02	0.19 ± 0.01	0.15 ± 0.09	−0.42	0.87**
	Ferulic acid	0.47 ± 0.04	0.31 ± 0.03	0.06 ± 0.04	0.16 ± 0.11	1.35	−0.61**
	Salicylic acid	2.28 ± 0.24	7.70 ± 1.42	0.02 ± 0.00	0.02 ± 0.02	0.39	1.76**
Flavonoids	Prunin	2.41 ± 0.51	5.61 ± 0.19	0.04 ± 0.01	0.02 ± 0.02	−0.82	1.22*
	Neohesperidin	0.17 ± 0.03	1.27 ± 0.24	0.20 ± 0.11	0.29 ± 0.38	0.57	2.90**
	Vanillin	0.01 ± 0.00	0.33 ± 0.26	0.73 ± 0.85	1.81 ± 0.84	1.32	5.36

**Note:**

The changes in gas exchange of the two soybean ecotypes young and old leaves. P, net photosynthetic rate; g, stomatal conductance; E, transpiration rate; C/C, ratio of sub-stomatal to atmospheric CO concentrations; GS1, common soybean; GS2, barren tolerant wild soybean. CK, control treatment; LN, low-nitrogen stress; one asterisk (*) indicates significance (< 0.05), two asterisks (**) indicate significance (p < 0.01).

Compared with CK, the relative concentrations of the amino acid metabolites L-proline, sarcosine, L-homoserine, and L-aspartic acid increased significantly 2.85-fold (*p* < 0.01), 1.88-fold (*p* < 0.05), 1.22-fold (*p* < 0.01), and 2.28-fold (*p* < 0.05), respectively in the old leaves of GS2, but the trend in old leaves of GS1 under LN conditions was decreased relative to CK (Table 4). It is noteworthy that the relative concentrations of GABA increased significantly 0.92-fold (*p* < 0.05) in GS2 old leaves. The relative concentrations of isoleucine increased significantly 1.79-fold (*p* < 0.01) in GS2 under LN, but no significant changes were found in the old leaves of GS1 (Table 4).

Under LN stress, the relative concentrations of organic acid metabolites related to glycolysis and the TCA pathway, including pyruvic acid, L-malic acid, and succinic acid, increased significantly 0.94-, 0.45-fold and 1.07-fold (*p* < 0.05), respectively, in GS1, and increased significantly 1.31-, 0.64-fold, and 0.67-fold (*p* < 0.05), respectively, in GS2 compared with CK. Fructose 6-phosphate decreased 0.72-fold (*p* < 0.05) in GS1, but increased significantly 1.25-fold (*p* < 0.01) in GS2. The relative concentrations of organic acids including glycolic acid, galactaric acid, benzoic acid, and glyceric acid increased significantly 1.25-fold, 1.26- fold, 3.37-fold, and 4.19-fold (*p* < 0.05) in GS1, whereas lactic acid, maleic acid, galactaric acid, and glyceric acid increased significantly 0.44- fold, 2.41-fold, 0.44-fold, and 0.67-fold (*p* < 0.05) in GS2. The relative content of shikimic acid increased in the old leaves of GS2 but no significant changes were observed in the old leaves of GS1 under LN stress. The relative concentrations of palmitic acid, stearic acid, pelargonic acid, and linoleic acid decreased significantly 1.20-fold (*p* < 0.05), 0.33-fold (*p* < 0.01), 0.22-fold (*p* < 0.01), and 0.15-fold (*p* < 0.01), respectively, in GS1 but increased significantly 1.59-fold (*p* < 0.05), 0.87-fold (*p* < 0.05), 0.27-fold (*p* < 0.05), and 1.41-fold (*p* < 0.01), respectively, in old leaves of GS2 under LN stress. The relative salicylic acid and prunin content increased significantly 1.76-fold (*p* < 0.01) and 1.22-fold (*p* < 0.05), respectively, in GS2 under LN (Fig. 4).

## Discussion

In the process of adapting to a stressful environment, wild soybean has evolved a large number of ecotypes exhibiting valuable characteristics (Lu et al., 2020). Leaves of different ages respond positively in plants that are tolerating adverse environments (Liu et al., 2019). In the current study, the mechanism of LN tolerance in a wild soybean ecotype was analyzed from the perspective of leaf nutrient reabsorption and we assessed the plants for morphometry and rhizobium formation under LN conditions. N deficiency inhibited the growth of the seedlings of both wild soybean ecotypes according to their different adaptation to LN stress. The biomass of the tolerant GS2 seedlings decreased less than the GS1 seedlings under LN conditions and physiological and metabolite regulation in GS2 may play an important role in the process of tolerating low-nitrogen stress.

In the present study, plant phenotypes and biomass accumulation further confirmed that LN stress could delay plant development, with GS2 exhibiting a greater LN tolerance. TC and TN data display a worse N deficiency in the young leaves of GS1than in GS2. N deficiency will destroy the chloroplast membrane and reduce chlorophyll content (Ohwaki & Sugahara, 1997). Under LN stress, photosynthetic pigment concentrations declined less in the young and old leaves of LN- tolerant GS2 than in GS1, compared with the CK. This helped GS2 to maintain relatively stable photosynthesis rates in both young and old leaves (Chen et al., 2019). The gas-exchange coefficient increased in both ecotypes, with the difference being more significant in the young leaves of GS2 than in GS1, compared with CK. The wild soybean younger leaves have higher *P*_N_ and *g*_*s*_ than old leaves, indicating that under low nitrogen stress, the carbon metabolism ability in the new leaves of wild soybean is stronger than that of the old leaves, which is a manifestation of its active resistance to low nitrogen stress. Indicating that GS2 slows down the photosynthetic rate in old leaves to reduce energy and material consumption to maintain relatively stable photosynthetic characteristics and resist LN stress. However, cultivated soybean younger leaves have lower *P*_N_ and *g*_*s*_ than old leaves, indicating an imbalance in their photosynthetic characteristics under low nitrogen stress.

It could be seen from the results that NO_3_^−^ deficiency in GS1 leaves was more severe than in GS2 leaves which indicated that the barren-tolerant GS2 ecotype was more efficient in the process of absorption and storage of N under LN. The SO_4_^2−^ concentrations decreased significantly in the old leaves of GS2 but increased in those of GS1. SO_4_^2−^ concentrations decreased more sharply in the young leaves of GS1 than in those of GS2. Sulfur is essential element in the active center of enzymatic reactions (Gao, Zhang & Ma, 2009) and it also plays a certain role in the process of plant growth regulation, detoxification, defense, and stress tolerance (Bollig et al., 2019). Organic sulfur can be converted into inorganic sulfur (mainly sulfate, SO_4_^2−^) by protein hydrolysis, sulfate can be converted into organic sulfur by metabolism, and can be transferred to young tissues for reuse (Liu et al., 2020). The results indicate that GS2 transferred most of the sulfate from senescent older leaves to young tender tissues to alleviate the obstruction by ion imbalance of plant growth. Mineral elements have a marked effect on the improvement of nitrogenase activity in the processes of nitrogen reduction and fixation (Lecourt et al., 2015). Previous studies have suggested that changes in the availability of one element could exert an effect on the uptake and accumulation of other elements in plants (Xue et al., 2012). In GS2 the concentrations of Mn^2+^ and Zn^2+^ in the young leaves, and Cl^−^, H_2_PO_4_^−^, Mn^2+^, B^3+^, Fe^2+^, and Zn^2+^ in the old leaves were particularly significant increased. Indicating beneficial mineral ions were enriched in the old leaves of GS2, which could help GS2 to avoid ion imbalance under LN stress (Zerche et al., 2016).

According to the results of the present study, the relative GABA concentrations decreased significantly in young leaves of GS1, although it increased in both the young and old leaves of GS2. GABA acts as an endogenous signaling molecule in plant growth and development and rapidly accumulates in plant tissue in response to several abiotic and biotic stresses (Nayyar et al., 2014; Priya et al., 2019). Firstly, under LN stress, hydroxyls produced by nitrate reduction decreased and the frequency of electron carriers in the electron transport chain decreased, resulting in a significant increase in ROS (Ramesh et al., 2015). GABA plays a crucial role in the scavenging of ROS generated rapidly due to disruption of the intracellular redox equilibrium and hence significantly mitigates oxidative stress-induced damages in plants (Streeter, Lohnes & Fioritto, 2001). Secondly, GABA has long been considered an important link in C and N metabolism since the GABA shunt pathway competes with the respiratory chain for SSADH (Shelp, Bown & McLean, 1999; Kinnersley & Turano, 2000). Glutamic acid, the precursor of GABA, is considered to be the main form of nitrogen accumulated in the roots of plants. Under an adverse environment, plants can make the nitrogen of glutamic acid flow into GABA and proline to regulate the metabolism of nitrogen, which is a key factor in the rapid response of plants to external stimuli (Nam et al., 2015; Shelp et al., 2012). Under salt stress, the accumulation of GABA in Arabidopsis leads to an increase in amino acids and the activity of glutamate dehydrogenase (GAD), increasing the expression of GAD instantaneously, and thus increasing the flux of the GABA shunt and other related pathways to regulate TC/TN balance (Renault et al., 2010). Under stress, the GABA shunt may perform a cataplerotic role by providing carbon to the cycle through the catabolism of GABA to succinic acid (Barbosa et al., 2010). Moreover, GABA is also a temporary N storage compound. The glutamate to GABA conversion is increased under conditions that inhibit glutamine synthesis, reduce protein synthesis, or enhance degradation (Sita & Kumar, 2020). Evidence also indicates that glutamine and GABA are produced during protein storage and mobilization as a means of recycling Arg-derived N and C (Shelp, Bown & McLean, 1999). Thus, the GABA shunt may be of considerable importance in the N economy of plants. Because, in the current study, the accumulation of GABA in the young and old leaves of GS2 was highly consistent, it can be inferred that GS2 can regulate TC/TN balance, antioxidants concentration, and N storage by enriching GABA content to alleviate stress (Kinnersley & Turano, 2016).

Asparagine has a higher N/C ratio than glutamine and can be used as a long-range transport and storage compound, especially in legumes (Masclaux-Daubresse et al., 2010; Lu et al., 2013). Under abiotic stress, the energy supply is insufficient, GS and Fd-GOGAT are inhibited, whereas AS is activated, and N assimilation proceeded toward asparagine metabolism. In the present study, the relative concentrations of asparagine increased in the old leaves of both GS1 and GS2 and decreased significantly in young leaves of only GS1, which indicates that N was transported and reused in GS2 but does not change significantly in young leaves of GS2. Earlier study had confirmed that increases in asparagine concentrations suggest that it will be easier to render available N for remobilization from senescing leaves (Tegeder & Masclaux-Daubresse, 2018). In the current study, the relative concentrations of L-homoserine and isoleucine increased significantly in young and old leaves of GS2. The effective transport and utilization of these key amino acids between young and old leaves provide a relatively stable material basis for protein synthesis in the vigorous young growth parts of GS2.

Organic acid metabolism is of fundamental importance at the cellular level for several biochemical processes including energy production, formation of precursors for amino-acid biosynthesis, and, at the whole plant level, modulation of adaptation to the environment (López-Bucio et al., 2000). Organic acids exuded by roots originate from the downward transport of organic acids in leaves (Hildebrandt et al., 2015). The present results showed that most differential organic acids (*e.g*., L-lactic acid, maleic acid, caffeic acid, galactaric acid, benzoic acid, and glyceric acid) increased significantly in the old leaves of GS2 but no significant change was found in the corresponding leaves of GS1. Organic acid metabolism not only provides carbon skeletons during N assimilation but also plays potential roles in osmotic regulation, cation balance, nutrient deficiency-related coping mechanisms, and plant–microbe interactions at the root–soil interface (Gregersen et al., 2013). Organic acids have also been found to stimulate nitrate uptake. Nitrate uptake rates by roots of intact soybean plants are stimulated by malate, which moves down the phloem and accumulates in the root (Touraine, Muller & Grignon, 1992). Some plants have specialized roots that exude large amounts of organic acids (corresponding to up to 23% of net photosynthesis), which acidify the soil and chelate metal ions around the roots, resulting in the mobilization of micronutrients (Schachtman & Shin, 2007).

Fritz et al. (2006) found that the levels of 2-oxoglutarate and other TCA cycle intermediates, including citrate, isocitrate, succinate, fumarate, and malate, which are key regulators of carbon–nitrogen interaction, were lower under LN conditions. In the present study, the results showed that, under LN conditions, most differential metabolites related to glycolysis and the TCA pathway did not change significantly in the young leaves of GS2 under LN stress. However, compared with CK, the differences in concentrations of metabolites associated with the energy consumption stage of glycolysis (fructose-6-phosphate) increased significantly in the young leaves of GS1, under LN stress. In the second stage of glycolysis (energy generation), the relative concentrations of organic metabolites of pyruvic acid decreased significantly in the young leaves of GS1 (Geigenberger et al., 1993). In the old leaves of GS2, in particular, the relative concentrations of glycolysis-related metabolites of fructose-6-phosphate and pyruvic acid increased significantly, indicating that the old leaves of GS2 strengthen and generate energy (Avila-Ospina, Clément & Masclaux-Daubresse, 2017). The increase of L-malic acid and succinic acid concentrations enhanced the TCA cycle in old leaves of GS2. Compared with GS1, the strategies of glycolysis and TCA in the young leaves of GS2 could conserve energy from old leaves and maintain a relatively stable state under LN stress (Huang et al., 2008).

## Conclusions

Improved carbon assimilation capacity and maintenance of adequate reabsorption of mineral and organic nutrients is an important survival strategy for barren-tolerant wild soybeans under low nitrogen stress. Compared with the common wild soybean, the barren-tolerant wild soybean has a relatively stable ability to maintain photosynthetic assimilation by young leaves and the supply of inorganic nutrients under low-nitrogen stress. In addition, the enrichment of specific nitrogen-containing compounds in old leaves of GS2, such as GABA and asparagine, can play a substantial role in N storage, TC/TN balance and antioxidant defense and can act as signaling molecules to help barren-tolerant soybean tolerate LN stress. The increase in the relative organic acid concentration in the old leaves of GS2 could improve the utilization rate of N in the soil. The rate of fatty acid catabolism and the TCA cycle in the old leaves of GS2 maintained a relatively high level of energy supply, provided an energy basis for nutrient transport and reuse, and improved the ability to tolerate low-nitrogen stress. Our study provides a quantitative standard by which to select, evaluate and utilize wild soybean germplasm accessions and provides an important scientific basis for research on plant divergent evolution.

## Supplemental Information

10.7717/peerj.15486/supp-1 Supplemental Information 1 Formulation of nutrient solution and low nitrogen stress treatment solution for plants growth.

10.7717/peerj.15486/supp-2 Supplemental Information 2 Biomass of two wild soybean ecotypes under control and LN stress. Values are presented as mean ± standard deviation of four biological replicates; GS1, common wild soybean; GS2, low nitrogen-tolerant wild soybean; CK, control treatment; LN, low nitrogen stress; Significant differences between controls and LN treatments were determined by the One-Way ANOVA test and marked as “*” (*p* < 0.05) and “**” (*p* < 0.01).

10.7717/peerj.15486/supp-3 Supplemental Information 3 The contributions of ions among young and old leaves of W1 and W2 seedlings to the first principal component (PC1) and the second principal component (PC2).

10.7717/peerj.15486/supp-4 Supplemental Information 4 The contributions of metabolites among young leaves of W and C seedlings to the first principal component (PC1) and the second principal component (PC2).

10.7717/peerj.15486/supp-5 Supplemental Information 5 The contributions of metabolites among old leaves of W and C seedlings to the first principal component (PC1) and the second principal component (PC2).

10.7717/peerj.15486/supp-6 Supplemental Information 6 Raw data of carbon and nitrogen contents in young and old leaves of two wild soybean varieties under LN stress.

10.7717/peerj.15486/supp-7 Supplemental Information 7 Raw data of the changes in gas exchange of the two soybean ecotypes young and old leaves.

10.7717/peerj.15486/supp-8 Supplemental Information 8 Raw data of the changes in photosynthetic pigment content of the two soybean ecotypes young and old leaves.

10.7717/peerj.15486/supp-9 Supplemental Information 9 Raw data of Ion contents in young and old leaves of two wild soybean ecotypes under LN stress.

10.7717/peerj.15486/supp-10 Supplemental Information 10 Raw data of changes of LN stress on metabolite content in young leaves of two wild soybean ecotypes.

10.7717/peerj.15486/supp-11 Supplemental Information 11 Raw data of changes of LN stress on metabolite content in old leaves of two wild soybean ecotypes.

10.7717/peerj.15486/supp-12 Supplemental Information 12 Total ion current chromatograms of common wild soybean extracts obtained from GC-MS. A: GS1-YL-CK; B: GS1-YL-LN; C: GS1-OL-CK; D: GS1-OL-LN

10.7717/peerj.15486/supp-13 Supplemental Information 13 Total ion current chromatograms of barren-tolerant wild soybean extracts obtained from GC-MS. A: GS2-YL-CK; B: GS2-YL-LN; C: GS2-OL-CK; D: GS2-OL-LN

## Additional Information and Declarations

### Data Availability

The following information was supplied regarding data availability: The raw measurements are available in the Supplemental Files.

## References

[ref-1] Abdurakhmonov IY, Abdusattor A (2008). Application of association mapping to understanding the genetic diversity of plant germplasm resources. International Journal of Plant Genomics.

[ref-2] Araújo WL, Tohge T, Ishizaki K, Leaver CJ, Fernie AR (2011). Protein degradation-an alternative respiratory substrate for stressed plants. Trends in Plant Science.

[ref-3] Arun M, Radhakrishnan R, Ai TN, Naing AH, Lee IJ, Kim CK (2016). Nitrogenous compounds enhance the growth of petunia and reprogram biochemical changes against the adverse effect of salinity. The Journal of Horticultural Science and Biotechnology.

[ref-4] Avila-Ospina L, Clément G, Masclaux-Daubresse C (2017). Metabolite profiling for leaf senescence in barley reveals decreases in amino acids and glycolysis intermediates. Agronomy.

[ref-5] Barbosa JM, Singh NK, Cherry JH, Locy RD (2010). Nitrate uptake and utilization is modulated by exogenous γ-aminobutyric acid in *Arabidopsis thaliana* seedlings. Plant Physiology and Biochemistry.

[ref-6] Bollig K, Zahn M, Myint SS, Hogekamp C, Küster H, Horst WJ (2019). Sulfur supply improves tomato pathogen resistance.

[ref-7] Chen JH, Hao ZD, Guang XM, Zhao CX, Wang PK, Xue LJ, Zhu QH, Yang LF, Sheng Y, Zhou YW, Xu HB, Xie HQ, Long XF, Zhang J, Wang ZG, Shi M, Lu Y, Liu S, Guan L, Zhu Q, Yang L, Ge S, Cheng T, Laux T, Gao Q, Peng Y, Liu N, Yang S, Shi J (2019). Liriodendron genome sheds light on angiosperm phylogeny and species-pair differentiation. Nature Plants.

[ref-54] Elger A, Lemoine DG, Fenner M, Hanley ME (2009). Plant ontogeny and chemical defence: older seedlings are better defended. Oikos.

[ref-8] Fritz C, Palacios-Rojas C, Feil R, Stitt M (2006). Regulation of secondary metabolism by the carbon-nitrogen status in tobacco: nitrate inhibits large sectors of phenylpropanoid metabolism. Plant Journal.

[ref-9] Galloway JN, Dentener FJ, Capone DG, Boyer EW, Howarth RW, Seitzinger SP, Asner GP, Cleveland CC, Green PA, Holland EA, Karl DM, Michaels AF, Porter JH, Townsend AR, Vorosmarty CJ (2004). Nitrogen cycles: past, present, and future. Biogeochemistry.

[ref-10] Gao SJ, Wang SY, Hu YN, Chen SY, Guo JX, Shi LX (2022). Comparative differences in photosynthetic characteristics, ion balance, and nitrogen metabolism between young and old wild soybean leaves under nitrogen deficiency. Plant Stress.

[ref-11] Gao ZC, Zhang XL, Ma XZ (2009). Research advances in physiological function of element sulphur in plants. Heilongjiang Agricultural Sciences.

[ref-12] Geigenberger P, Langenberger S, Wilke I, Heineke D, Heldt HW, Stitt M (1993). Sucrose is metabolised by sucrose synthase and glycolysis within the phloem complex of *Ricinus communis* L. seedlings. Planta.

[ref-13] Gregersen PL, Culetic A, Boschian L, Krupinska K (2013). Plant senescence and crop productivity. Plant Molecular Biology.

[ref-17] Häusler RE, Ludewig F, Krueger S (2014). Amino acids-a life between metabolism and signaling. Plant Science.

[ref-14] Hildebrandt TM, Nunes Nesi A, Araújo WL, Braun HP (2015). Amino acid catabolism in plants. Molecular Plant.

[ref-15] Holm G (1954). Chlorophyll mutation in barley. Acta Agriculturae Scandinavica.

[ref-16] Huang CY, Roessner U, Eickmeier I, Genc Y, Callahan DL, Shirley N, Langridge P, Bacic A (2008). Metabolite profiling reveals distinct changes in carbon and nitrogen metabolism in phosphate-deficient barley plants (*Hordeum vulgare* L.). Plant and Cell Physiology.

[ref-18] Jiao Y, Bai ZZ, Xu JS, Zhao ML, Khan Y, Hu YJ, Shi LX (2018). Metabolomics and its physiological regulation process reveal the salt-tolerant mechanism in *Glycine soja* seedling roots. Plant Physiology and Biochemistry.

[ref-55] Kichey T, Hirel B, Heumez E, Dubois F, Le Gouis J (2007). In winter wheat (*Triticum aestivum* L.), post-anthesis nitrogen uptake and remobilisation to the grain correlates with agronomic traits and nitrogen physiological markers. Field Crops Research.

[ref-19] Kinnersley AM, Turano FJ (2000). Gamma aminobutyric acid (GABA) and plant responses to stress. Critical Reviews in Plant Sciences.

[ref-20] Kinnersley AM, Turano FJ (2016). Gamma aminobutyric acid (GABA) and Plant responses to stress. Critical Reviews in Plant Sciences.

[ref-21] Lecourt J, Lauvergeat V, Ollat N, Vivin P, Cookson SJ (2015). Shoot and root ionome responses to nitrate supply in grafted grapevines are rootstock genotype dependent. Australian Journal of Grape and Wine Research.

[ref-22] Li MX, Guo R, Jiao Y, Jin XF, Zhang HY, Shi LX (2017). Comparison of Salt tolerance in *Soja* based on metabolomics of seedling roots. Frontiers in Plant Science.

[ref-23] Liu DP, Li MX, Liu Y, Shi LX (2020). Integration of the metabolome and transcriptome reveals the resistance mechanism to low nitrogen in wild soybean seedling roots. Environmental and Experimental Botany.

[ref-24] Liu Y, Li M, Xu J, Liu X, Wang S, Shi LX (2019). Physiological and metabolomics analyses of young and old leaves from wild and cultivated soybean seedlings under low-nitrogen conditions. BMC Plant Biology.

[ref-28] López-Bucio J, Nieto-Jacobo MF, Ramírez-Rodríguez V, Herrera-Estrella L (2000). Organic acid metabolism in plants: from adaptive physiology to transgenic varieties for cultivation in extreme soils. Plant Science.

[ref-25] Lu SJ, Dong L, Fang C, Liu S, Kong L, Cheng Q, Chen L, Su T, Nan H, Zhang D, Zhang L, Wang Z, Yang Y, Yu D, Liu X, Yang Q, Lin X, Tang Y, Zhao X, Yang X, Tian C, Xie Q, Li X, Yuan X, Tian Z, Liu B, Weller J, Kong F (2020). Stepwise selection on homeologous PRR genes controlling flowering and maturity during soybean domestication. Nature Genetics.

[ref-26] Lu Y, Lam H, Pi E, Zhan Q, Tsai S, Wang C, Kwan Y, Ngai S (2013). Comparative metabolomics in *Glycine max* and *Glycine soja* under salt stress to reveal the phenotypes of their offspring. Journal of Agricultural and Food Chemistry.

[ref-27] Luca GAD, Mufarrege MM, Hadad HR, Maine MA (2019). Nitrogen and phosphorus removal and *Typha domingensis* tolerance in a floating treatment wetland. The Science of the Total Environment.

[ref-29] Masclaux-Daubresse C, Daniel-Vedele F, Dechorgnat J, Chardon F, Gaufichon L, Suzuki A (2010). Nitrogen uptake, assimilation and remobilization in plants: challenges for sustainable and productive agriculture. Annals of Botany.

[ref-30] Mei HS, Thimann KV (1984). The relation between nitrogen deficiency and leaf senescence. Physiologia Plantarum.

[ref-31] Mourtzinis S, Kaur G, Orlowski JM, Shapiro CA, Lee CD, Wortmann C, Holshouser D, Nafziger ED, Kandel H, Niekamp J, Ross WJ, Lofton J, Vonk J, Roozeboom KL, Thelen KD, Lindsey LE, Staton M, Naeve SL, Casteel SN, Wiebold WJ, Conley SP (2018). Soybean response to nitrogen application across the United States: a synthesis-analysis. Field Crops Research.

[ref-32] Nam MH, Bang E, Kwon TY, Kim Y, Kim EH, Cho K, Park WJ, Kim BG, Yoon IS (2015). Metabolite profiling of diverse rice germplasm and identification of conserved metabolic markers of rice roots in response to long-term mild salinity stress. International Journal of Molecular Sciences.

[ref-33] Nayyar H, Kaur R, Kaur S, Singh R (2014). γ-Aminobutyric acid (GABA) imparts partial protection from heat stress injury to rice seedlings by improving leaf turgor and upregulating osmoprotectants and antioxidants. Journal of Plant Growth Regulation.

[ref-57] Ohwaki Y, Sugahara K (1997). Active extrusion of protons and exudation of carboxylic acids in response to iron deficiency by roots of chickpea (Cicer arietinum L.). Plant and Soil.

[ref-34] Priya M, Sharma L, Kaur R, Bindumadhava H, Nair RM, Siddique KHM, Nayyar H (2019). GABA (γ-aminobutyric acid), as a thermo-protectant, to improve the reproductive function of heat-stressed mungbean plants. Scientific Reports.

[ref-35] Ramesh S, Tyerman S, Xu B, Bose J, Kaur S, Conn V, Gonçalves P, Lehre S, Wege S, Shabala S, Feijó J, Ryan P, Gilliham M (2015). Corrigendum: GABA signalling modulates plant growth by directly regulating the activity of plant-specific anion transporters. Nature Communications.

[ref-36] Renault H, Roussel V, Amrani A, Arzel M, Renault D, Bouchereau A, Deleu C (2010). The *Arabidopsis* pop2-1mutant reveals the involvement of GABA transaminase in salt stress tolerance. BMC Plant Biology.

[ref-37] Schachtman DP, Shin R (2007). Nutrient sensing and signaling: NPKS. Annual Review of Plant Biology.

[ref-38] Shelp BJ, Bown AW, McLean MD (1999). Metabolism and functions of gamma-aminobutyric acid. Trends in Plant Science.

[ref-39] Shelp BJ, Bozzo GG, Trobacher CP, Zarei A, Deyman KL, Brikis CJ (2012). Hypothesis/review: contribution of putrescine to 4-aminobutyrate (GABA) production in response to abiotic stress. Plant Science.

[ref-40] Shi LX, Ma S, Fang Y, Xu J (2015). Crucial variations in growth and ion homeostasis of *Glycine gracilis* seedlings under two types of salt stresses. Journal of Soil Science and Plant Nutrition.

[ref-41] Sita K, Kumar V (2020). Role of gamma amino butyric acid (GABA) against abiotic stress tolerance in legumes: a review. Plant Physiology Reports.

[ref-42] Streeter JG, Lohnes DG, Fioritto RJ (2001). Patterns of pinitol accumulation in soybean plants and relationships to drought tolerance. Plant Cell and Environment.

[ref-43] Sun Q, Lu HR, Zhang Q, Wang D, Chen J, Xiao JL, Ding XD, Li Q (2021). Transcriptome sequencing of wild soybean revealed gene expression dynamics under low nitrogen stress. Journal of Applied Genetics.

[ref-56] Taylor L, Nunes-Nesi A, Parsley K, Leiss A, Hibberd JM (2010). Cytosolic pyruvate, orthophosphate dikinase functions in nitrogen remobilization during leaf senescence and limits individual seed growth and nitrogen content. The Plant Journal: For Cell and Molecular Biology.

[ref-44] Tegeder M, Masclaux-Daubresse C (2018). Source and sink mechanisms of nitrogen transport and use. New Phytologist.

[ref-45] Touraine B, Muller B, Grignon C (1992). Effect of phloem-translocated malate on NO(3)-uptake by roots of intact soybean plants. Plant Physiology.

[ref-46] Wang H, Guo R, Hu YJ, Han DF (2019). Carbon-nitrogen metabolic responses and adaptive strategies to low-nitrogen stress in *Glycine soja*. Notulae Botanicae Horti Agrobotanici.

[ref-60] Wei YH, Shi AB, Jia XT, Zhang ZY, Ma XM, Gu MX, Meng XD, Wang XC (2018). Nitrogen supply and leaf age affect the expression of Ta GS1 or TaGS2 driven by a constitutive promoter in transgenic tobacco. Genes.

[ref-47] Xia J, Mandal R, Sinelnikov IV, Broadhurst D, Wishart DS (2012). MetaboAnalyst 2.0-a comprehensive server for metabolomic data analysis. Nucleic Acids Research.

[ref-48] Xie M, Chung CY, Li M, Wong F, Wang X, Liu A, Wang ZL, Leung AK, Wong T, Tong S, Xiao ZX, Fan KJ, Ng M, Qi XP, He JX, Chan T, Lam H (2019). A reference-grade wild soybean genome. Nature Communications.

[ref-49] Xue YF, Yue SC, Zhang YQ, Cui ZL, Chen XP, Yang FC, Cakmak I, McGrath SP, Zhang FS, Zou CQ (2012). Grain and shoot zinc accumulation in winter wheat affected by nitrogen management. Plant and Soil.

[ref-50] Yi LZ, Dong NP, Yun YH, Deng BC, Ren DB, Liu S, Liang YZ (2016). Chemometric methods in data processing of mass spectrometry-based metabolomics: a review. Analytica Chimica Acta.

[ref-51] Zerche S, Haensch KT, Druege U, Hajirezaei MR (2016). Nitrogen remobilisation facilitates adventitious root formation on reversible dark-induced carbohydrate depletion in *Petunia hybrida*. BMC Plant Biology.

[ref-52] Zhang HY, Mittal N, Leamy LJ, Barazani O, Song BH (2017). Back into the wild-apply untapped genetic diversity of wild relatives for crop improvement. Evolutionary Applications.

[ref-53] Zhao ML, Guo R, Li MX, Liu Y, Shi LX (2019). Physiological characteristics and metabolomics reveal the tolerance mechanism to low nitrogen in *Glycine soja* leaves. Physiologia Plantarum.

