# Supplementary figures and images for: Comparative study of leaf nutrient reabsorption by two different ecotypes of wild soybean under low-nitrogen stress
Source: PeerJ. 2023 Jun 27;11:e15486. doi: 10.7717/peerj.15486 (PMC10312162; doi:10.7717/peerj.15486)

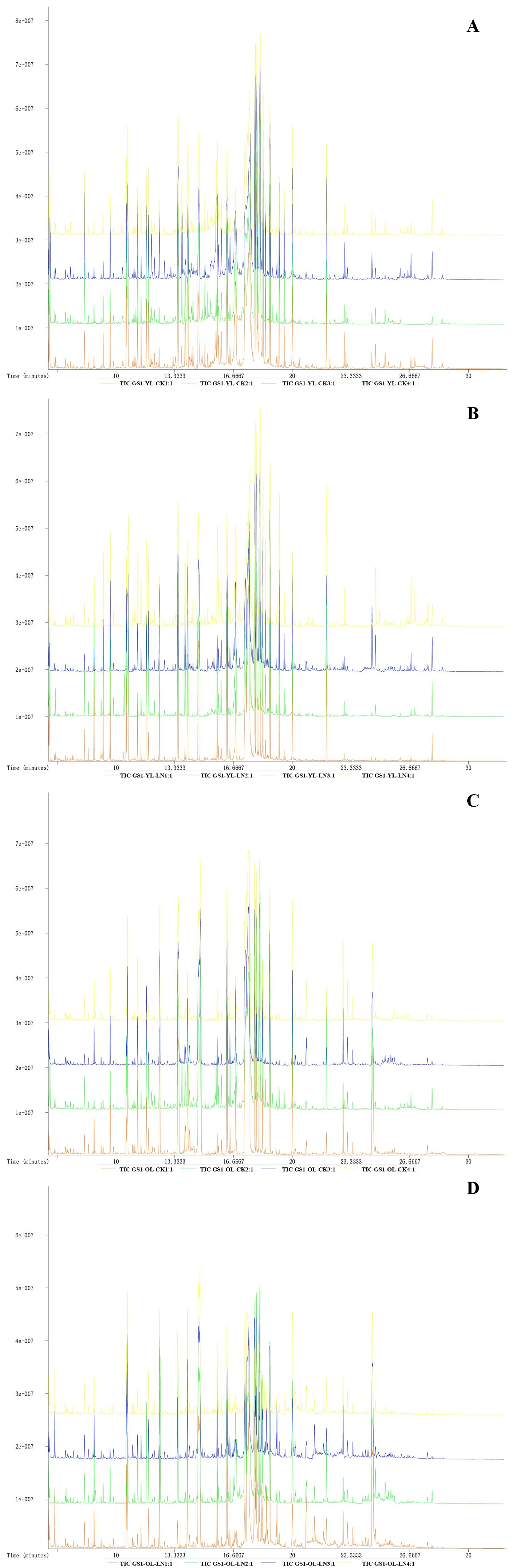

Supplement: Supplemental Information 12 — A: GS1-YL-CK; B: GS1-YL-LN; C: GS1-OL-CK; D: GS1-OL-LN [file peerj-11-15486-s012.jpg]

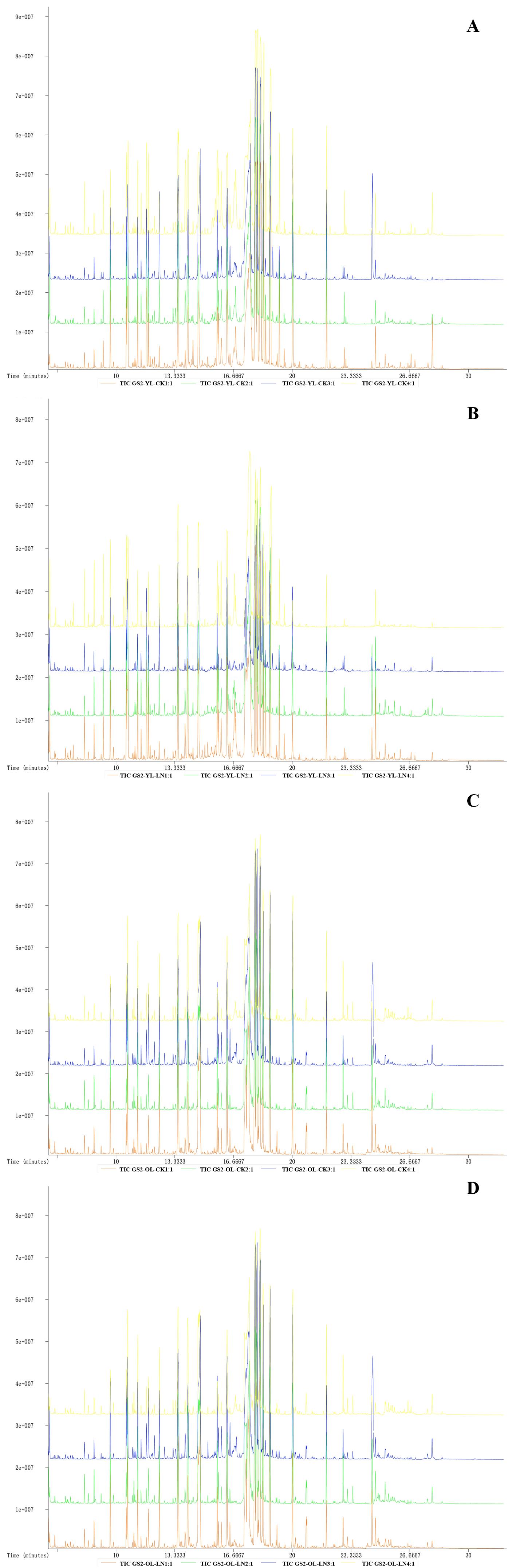

Supplement: Supplemental Information 13 — A: GS2-YL-CK; B: GS2-YL-LN; C: GS2-OL-CK; D: GS2-OL-LN [file peerj-11-15486-s013.jpg]
